# Evaluation on blood coagulation and C-reactive protein level among children with mycoplasma pneumoniae pneumonia by different chest imaging findings

**DOI:** 10.1097/MD.0000000000023926

**Published:** 2021-01-22

**Authors:** Juan Wang, Jianping Mao, Gang Chen, Yuanmei Huang, Jinjin Zhou, Changlong Gao, Danting Jin, Chenying Zhang, Juan Wen, Jun Sun

**Affiliations:** a Department of Pediatrics; b Department of Hematology; c Department of Medical Laboratory; d Department of Respiratory, the First People's Hospital of Lianyungang, Lianyungang, China; e Nanjing Maternity and Child Health Care Institute, Women's Hospital of Nanjing Medical University, Nanjing Maternity and Child Health Care Hospital, Nanjing, China.

**Keywords:** blood coagulation, chest imaging findings, C-reactive protein, mycoplasma pneumonia

## Abstract

Mycoplasma pneumoniae infection may induce a systemic hypercoagulable abnormality, like organ embolism and infarction. Indexes of blood coagulation and C-reactive protein (CRP) have been reported different between healthy people and mycoplasma pneumoniae pneumonia (MPP) patients, but this difference in MPP patients with different chest imaging findings has rarely been reported.

We performed a retrospective study of 101 children with MPP and 119 controls, combined with radiological examination and blood tests, to compare the blood coagulation and CRP level among MPP children with different chest imaging findings.

For the MPP children with different chest imaging findings, there were significant differences in CRP, fibrinogen (FIB) and D-dimer (D-D) levels among subgroups (*P* = .004, *P* = .008 and *P* < .001 respectively). The CRP level in group of interstitial pneumonia was significantly higher than that in groups of bronchopneumonia and hilar shadow thickening (*P* = .003 and *P* = .001 respectively). And the FIB and D-D values in group of lung consolidation were significantly higher than that in the other 3 groups (all *P* < .05). When compared with controls, the white blood cell, CRP, FIB, and D-D levels in MPP children were significantly higher, and the activated partial thromboplastin time and thrombin time levels were significantly lower (all *P* < .05).

Our results showed that CRP level changed most significantly in group of interstitial pneumonia, whereas FIB, D-D levels changed most significantly in the lung consolidation group.

## 1 Introduction

Community-acquired pneumonia (CAP) is a common and sometimes lethal infection. Although its therapeutic options are multiplying, CAP remains a significant cause of morbidity and mortality among children worldwide. *Mycoplasma pneumoniae* (MP) is 1 of the most prevalent pathogens causing CAP in children.^[1–3]^ Infection by MP accounts for 15% to 20% of all CAP cases worldwide.^[4]^ Epidemiological studies in Japan have demonstrated that *MP* is the second powerful cause of CAP, just next to streptococcus pneumoniae .^[5]^

MP binds to the cilia with the help of P1 protein, multiplies in the respiratory epithelial layer,^[6]^ and stimulates the respiratory tract to produce proinflammatory cytokines such as IL-6, C-reactive protein (CRP), a process leading to acute cellular inflammatory reaction and airway damage.^[7]^ Hence, the main affected organ is lung. MP pneumonia (MPP) is clinically characterized by dry cough, fever and general fatigue. The chest imaging of MPP children include consolidation, atelectasis, ground glass opacity and pleural effusion.^[8]^

Nowadays, concerns have been raised about the effect of MP infection on extrapulmonary systems in children, including cardiovascular, neurologic, hematologic, dermatologic and hepatobiliary systems.^[9]^ In some cases, these effects can bring about severe, even life-threatening pneumonia. Koletsky and Weinstein reviewed eleven cases of fatal MP infection,^[10]^ only to find 1 with pneumonia. Surprisingly, abnormal blood coagulation and vascular thrombosis developed in 5 patients. Thus, we speculated that MP infection may induce a systemic hypercoagulable abnormality, like organ embolism and infarction. These cases have been reported recently, such as cerebral infarction, pulmonary embolism and splenic infarct.^[11,12]^ Indexes of blood coagulation and CRP have been reported different between healthy people and MPP patients,^[13]^ but this difference in MPP patients with different chest imaging findings has rarely been reported. Therefore, we performed a retrospective study of 101 children with MPP and 119 controls, combined with radiological examination and blood tests, to compare the blood coagulation and CRP level among MPP children with different chest imaging findings. This study may help physicians, especially the primary care physicians, to estimate the patients’ blood coagulation state in time, and give early intervention to prevent severe complications.

## 2 Subjects and methods

This study was conducted in the pediatric department of the First People's Hospital of Lianyungang (Jiangsu Province, China) from January 1, 2018 to December 31, 2018. The study was approved by the ethics committee of the hospital. All children's parents signed an informed consent form.

The inpatient children who showed symptoms (such as cough, fever, abnormal lung sounds, etc), abnormal chest X-ray images, invalid β-lactam antibiotics treatment, and positive anti-MP IgM specific antibody were included in this study. All subjects were divided into 4 subgroups based on different chest imaging findings: lung consolidation, interstitial pneumonia, bronchopneumonia and hilar shadow thickening.^[8]^ We excluded children if they fit the following criteria:

(1) Children with haematological diseases or coagulant functional abnormality;
(2) Children who took medicine that could affect the coagulation before admission
(3) Children with a history of trauma or surgery
(4) Children with lung malformation and diseases such as asthma;
(5) Children with serious cardiac, hepatic and renal insufficiency;
(6) Children with incomplete clinical data;
(7) Children with other infections;
(8) Children whose chest images were complex and varied as the disease continued.

At 7–10 days after pneumonia symptoms appeared, 2 mL of venous blood was collected. With this blood, 0.1 ml of serum was extracted, centrifuged, and transferred into reagent strips. Quantitative enzyme-linked immunosorbent assays were established to measure the levels of anti-MP IgM antibodies using IgM enzyme-linked immunosorbent assays kits (YHLO BIOTECH CO, LTD, Shenzhen China). The assay was considered positive if the level of IgM ≥ 1.1 mg.

For each child with MPP, 2 mL of venous blood was collected and sampled using BD tube (America) before treatment. After an interval of 60 minute, the blood sample was centrifuged at 3000 r/min for 5 min in a Xiang Yi L535–1 centrifuge (Xiang Yi laboratory instrument development co, LTD, Hunan, China) and the plasma was immediately separated. During the following 2 h, the coagulation status was evaluated with several indexes detected by the ACLTOP700 automatic blood coagulation analyser (Beckman Coulter), including prothrombin time (PT), activated partial thromboplastin time (APTT), thrombin time (TT), plasma fibrinogen (FIB) and D-dimer (D-D) levels. Other clinical indexes, such as white blood cell, CRP, platelet count, alanine transaminase (ALT) were analyzed with routine methods.

Meanwhile, 119 non-infectious inguinal hernia patients in pediatric surgery department of our hospital during the corresponding period were enrolled as the control group. The exclusion criteria were the same as that of the MPP group. Measurement of blood coagulation and anti-MP IgM antibody were conducted before surgery. Anti-MP IgM antibody in the control group was assured negative.

SPSS 20.0 (IBM Corp, Armonk, NY) was used for statistical analysis. Continuous data were shown as mean ± standard deviation (x ± s). All continuous data were tested for normality and homogeneity of variance. Analysis of variance or the Student *t*-test was used to determine differences of continuous variables between the groups. Least Significance Difference -*t* test method was used to identify significant differences between group means. Categorical data were shown as frequencies. Pearson chi-square test was used to analyze differences between categorical variables. Correlation analysis was used to detect the relationship between indexes. *P* < .05 was considered statistically significant.

## 3 Results

In total, 39 children were excluded due to ineligibility. Finally, 101 children with MPP (aged from 9 months to 12 years) and 119 controls (aged from 3 years to 14 years) were included in this study. Chest imaging findings showed that 17 cases were lung consolidation, 17 cases were interstitial pneumonia, 46 cases were bronchopneumonia, and 21 cases were hilar shadow thickening. The general characteristics and laboratory tests of children with MPP and controls are shown in Table 1. For the MPP children with different chest imaging findings, there were significant differences in age, height, weight and CRP levels among subgroups (*P* = 0.001, *P* = .007, *P* < .001 and *P* = .004 respectively). When compared with controls, the white blood cell and CRP levels in MPP children were significantly higher (*P* = .007 and *P* < .001 respectively). However, no significant differences were found in platelet and ALT between groups.

**Table 1:** The general characteristics and laboratory tests of children with mycoplasma pneumoniae pneumonia and controls.

	Cases							
Variables	Lung consolidation (n = 17)	Interstitial pneumonia (n = 17)	Bronchopneumonia (n = 46)	Hilar shadow thickening (n = 21)	All cases (n = 101)	Controls (n = 119)	*P*^1^	*P*^2^
Gender (boy)	9 (52.9%)	9 (52.9%)	21 (45.7%)	15 (71.4%)	54 (53.5%)	77 (64.7%)	.090	.277
Age (yr)	6.59 ± 3.46	3.99 ± 1.81	4.58 ± 2.14	3.53 ± 1.50	4.60 ± 2.43	5.77 ± 2.50	.001	.001
Height (cm)	119.59 ± 23.94	105.53 ± 13.65	108.57 ± 17.65	99.57 ± 12.92	108.04 ± 18.28	117.29 ± 17.65	<.001	.007
Weight (kg)	26.18 ± 11.80	16.47 ± 4.11	19.14 ± 7.19	16.71 ± 4.85	19.37 ± 7.99	22.27 ± 8.70	.011	<.001
WBC (10^^^9/L)	9.86 ± 3.93	10.36 ± 5.34	8.96 ± 3.45	8.61 ± 4.42	9.27 ± 4.09	8.02 ± 2.29	.007	.501
PLT (10^^^9/L)	277.94 ± 76.24	232.94 ± 76.14	257.57 ± 85.45	236.05 ± 74.96	252.38 ± 80.69	268.39 ± 65.74	.112	.293
CRP (mg/L)	21.90 ± 18.02	34.62 ± 54.82	12.31 ± 13.84	6.18 ± 6.87	16.40 ± 26.83	5.42 ± 9.77	<.001	.004
ALT (U/L)	22.82 ± 20.33	14.12 ± 4.69	16.82 ± 14.43	15.38 ± 5.00	17.08 ± 13.26	19.49 ± 8.96	.112	.222

*P*^1^ value for the comparison of all cases with controls; *P*^2^ value for the comparison among children with mycoplasma pneumoniae pneumonia by different chest imaging findings; ALT = alanine transaminase, CRP = C-reactive protein, PLT = platelet, WBC = white blood cell.

Then we compared the blood coagulation indexes among MPP children and controls (Table 2). For the MPP children with different chest imaging findings, there were significant differences in FIB and D-D levels among subgroups (*P* = .008 and *P* < .001 respectively). Slightly difference in TT levels was also observed among subgroups (*P* = .089). When compared with controls, the FIB and D-D levels in MPP children were significantly higher (all *P* < .001), and the APTT and TT levels were significantly lower (*P* = .003 and *P* = .029 respectively).

**Table 2:** Comparison of blood coagulation among children with mycoplasma pneumoniae pneumonia and controls.

	Cases							
Coagulation indexes	Lung consolidation (n = 17)	Interstitial pneumonia (n = 17)	Bronchopneumonia (n = 46)	Hilar shadow thickening (n = 21)	All cases (n = 101)	Controls (n = 119)	*P*^1^	*P*^2^
PT (s)	12.49 ± 1.33	12.02 ± 1.24	12.31 ± 1.13	12.57 ± 1.62	12.35 ± 1.29	12.23 ± 0.87	.446	.584
APTT (s)	31.11 ± 7.18	33.37 ± 4.19	33.22 ± 5.57	34.23 ± 4.18	33.10 ± 5.43	35.06 ± 4.11	.003	.358
FIB (g/L)	4.37 ± 0.86	3.69 ± 1.08	3.54 ± 0.81	3.46 ± 0.94	3.69 ± 0.94	2.44 ± 0.47	<.001	.008
TT (s)	13.45 ± 1.13	14.52 ± 1.84	14.39 ± 1.42	14.56 ± 1.59	14.29 ± 1.52	17.38 ± 15.20	.029	.089
D-D (mg/L)	600.18 ± 625.76	248.12 ± 177.38	186.11 ± 140.69	176.1 ± 116.00	264.16 ± 320.86	74.06 ± 53.16	<.001	<.001

*P*^1^ value for the comparison of all cases with controls; *P*^2^ value for the comparison among children with mycoplasma pneumoniae pneumonia by different chest imaging findings; APTT = activated partial thromboplastin time, D-D = D-dimer, FIB = plasma fibrinogen, PT = prothrombin time, TT = thrombin time.

Results of further multiple comparisons of CRP, FIB and D-D levels among MPP children with different chest imaging findings are shown in Table 3. The CRP level in group of interstitial pneumonia was significantly higher than that in groups of bronchopneumonia and hilar shadow thickening (*P* = .003 and *P* = .001 respectively). And the FIB and D-D values in group of lung consolidation were significantly higher than that in the other three groups (all *P* < .05). However, these 2 values showed no difference among the other 3 groups, including groups of interstitial pneumonia, bronchopneumonia, hilar shadow thickening (all *P* > .05).

**Table 3:** Multiple comparisons of CRP, FIB, and D-dimer in case groups.

Comparison	CRP (mg/L)	FIB (g/L)	D-D (mg/L)
Lung consolidation vs Interstitial pneumonia	0.149	0.031	0.001
Lung consolidation vs Bronchopneumonia	0.187	0.002	< 0.001
Lung consolidation vs Hilar shadow thickening	0.061	0.002	< 0.001
Interstitial pneumonia vs Bronchopneumonia	0.003	0.538	0.447
Interstitial pneumonia vs Hilar shadow thickening	0.001	0.420	0.442
Bronchopneumonia vs Hilar shadow thickening	0.363	0.737	0.894

All values were *P* values for LSD-*t* test. CRP = C-reactive protein, D-D = D-dimer, FIB = plasma fibrinogen.

Furthermore, correlations between CRP, ALT, anti-MP IgM and blood coagulation indexes (PT, APTT, FIB, TT and D-D ) were also analyzed (Table 4). Among MPP children, CRP level was correlated with PT, FIB and TT; ALT level was correlated with APTT and D-D; whereas anti-MP IgM was correlated with APTT (all *P* < .05). Among all subjects (MPP children and controls), CRP level was correlated with PT, FIB, and D-dimer; whereas ALT level was correlated with D-D (all *P* < .05).

**Table 4:** Correlations (r value) between CRP, ALT, Anti-MP IgM and blood coagulation indexes.

Indexes	PT (s)	*P*	APTT (s)	*P*	FIB (g/L)	*P*	TT (s)	*P*	D-D (mg/L)	*P*
CRP (mg/L, in all cases)	0.245	.014	−0.103	.305	0.355	<.001	−0.223	.025	0.150	.134
CRP (mg/L, in all subjects)	0.186	.006	−0.118	.080	0.390	< .001	−0.074	.275	0.226	.001
ALT (U/L, in all cases)	−0.054	.595	−0.203	.043	0.070	.491	−0.103	.307	0.602	<.001
ALT (U/L, in all subjects)	−0.013	.849	−0.123	.069	−0.020	.766	−0.013	.847	0.399	< .001
Anti-MP IgM (U/mL, in all cases)	−0.048	.634	−0.221	.027	−0.072	.475	0.107	.288	−0.001	.994

All subjects refer to all cases (children with mycoplasma pneumoniae pneumonia) and controls; ALT = alanine transaminase, APTT = activated partial thromboplastin time, CRP = C-reactive protein, D-D = D-dimer, FIB = plasma fibrinogen, PT = prothrombin time, TT = thrombin time.

## 4 Discussion

Unlike *S pneumoniae* that may directly infect the alveolar lumen, MP can infect the entire airway, even the interstitial lung and alveoli. MP infection may cause acute bronchiolitis with edema, bronchial wall ulcer, and perivascular or peribronchial interstitial opacities, including lymphocytes, plasma cells and macrophages.^[14,15]^ Given these differences in MP infection at different regions, the imaging findings of MPP may vary.^[16]^ MPP has various extrapulmonary manifestations. Among them, abnormal blood coagulation is drawing increasing attention in recent years. Also, the relationship between blood coagulation indexes and pro-inflammatory factors such as IL-6 and CRP, has also become a research focus.^[17,18]^ In this study, we found significant differences in CRP and blood coagulation indexes among groups with different chest imaging findings. These results suggested that pulmonary and extrapulmonary manifestations might be closely related and could be shown by some indexes. Our aim was to find the relationship between chest imaging results and blood coagulation. Once this relationship is clarified, clinicians can simply know whether the patients have the risk of hemagglutination or even embolism according to the manifestation of chest X-ray, and take relevant examination and intervention treatment as early as possible to reduce the occurrence of serious complications and improve the long-term prognosis. However, as none of the patients have thrombosis or embolism in the study, our results do not show that CRP and blood coagulation indexes are related with thrombosis and embolism. Thus, further studies on these correlations are needed.

In this study, the age, height and weight of children with lung consolidation were significantly higher than those of other subgroups. This phenomenon can be often encountered in clinical practice. MP is a culprit of respiratory infection in school-age children and adolescents. MP infection occurs to 40% of CAP children over 5 years.^[7]^ Meanwhile, chest radiograph can detect severer changes in older MPP children.^[19]^

Hypercoagulability caused by MP infection is not rare. It is considered that abnormal coagulation after MP infection is associated with inflammation, immune-mediated damage, anticoagulant protein inhibition, as well as liver damage. MP infection produces inflammatory factors (such as TNF-α, IL-6, IL-1β, etc) and increases CRP level.^[20]^ These inflammatory mediators damage vascular endothelial cells and activate the coagulation system, thereby cause microvascular dysfunction and thrombosis, even multiple organ failure. In the meantime, in severe MPP, the cell-mediated immune response is exaggerated and interleukin levels are elevated, resulting in diffuse alveolar damage with fibrinous exudates inside alveolar lumens that appear as consolidation on chest images.^[21–23]^ Several studies noted that children with severe symptoms were likely to show more consolidated features and more extrapulmonary manifestations including hypercoagulability.^[24–26]^ Cell-mediated immune response plays an important role in the development of MPP.^[27]^ Thereby, we speculate that the immune response may cause chest radiographic changes in children with hypercoagulability. From our results of correlation analysis, PT, D-D and FIB titers were correlated with CRP value. And there are also significant differences in CRP level between groups with different chest imaging findings. Thereby, CRP may play a crucial role in hypercoagulability caused by MP infection.

CRP, a plasma protein synthesized by the liver, is a highly sensitive and dynamic systemic marker for infection, inflammation and tissue damage.^[28]^ The immunoassay for CRP is of great clinical utility. Evidences and reports suggested that CRP as a pro-inflammatory factor, can act on endothelial cells, influence fibrinogen concentration and then result in acute thrombosis and chronic atherosclerosis.^[29]^ Some autologous ligands recognized by CRP overlap those by antiphospholipid (aPL) autoantibodies that are closely associated with hypercoagulation.^[28]^ Many studies have demonstrated a predictive relationship between increased CRP level and increased incidence of stroke, coronary and peripheral arterial disease.^[30–32]^ Libby et al have also found that people with high CRP level are more likely to develop stroke.^[33]^ Previous meta-analyses have also suggested that elevated CRP level is independently associated with high ischemic stroke risk.^[34,35]^ Therefore, we speculate that CRP may affect blood coagulation through multiple channels, although the exact mechanism is unclear.

As we know, immunologic mechanisms, such as formation of immune complexes or autoimmunity, hide behind vascular occlusion caused by MP infection. Transient aPL antibodies have been detected in many infectious diseases, including those caused by MP.^[36]^ Witmer reported that MP, splenic infarct and transient aPL antibodies may have a new association between each other.^[12]^ APL antibodies, like lupus anticoagulant or anticardiolipin antibodies, are a group of heterogeneous antibodies that direct against plasma proteins via binding to phospholipid surfaces. APL antibodies participate in the pathogenesis of recurrent thrombosis, usually termed as antiphospholipid syndrome. Thus, we hypothesize that aPL induced by immune response after MP infection may result in temporary hypercoagulation. Meanwhile, in this study, anti-MP IgM was correlated with APTT. It is indicated that immune response may trigger hypercoagulability. Furthermore, cell-mediated immunity or predominant response can be manifested by chest images.^[37]^ Thereby, we speculate that hypercoagulability and chest images may be linked together through immune response.

Liver damage was common in children with MP infection. In several reports, MP infection transiently influenced the activity of many coagulation factors, including II, VIII, or X.^[38]^ One possible mechanism behind this phenomenon is the consumptive coagulopathy due to hepatic deterioration. As a main inhibitor of thrombin, antithrombin III plays a protective role in the coagulation system.^[39]^ Liver dysfunction decreases the amount of released antithrombin III or induces its aberrant synthesis. In this study, although no significant difference in ALT level was found between groups, ALT level was significantly correlated with APTT and D-D.

In summary, inflammatory response and immune regulation induced by MP infection may cause abnormal chest X-ray images and blood coagulation. During MP infection, autoimmunity is damaged and inflammatory mediators are released. These mediators can change chest X-ray images and hemagglutination indexes.

## 5 Conclusions

Taken together, our results showed that CRP level changed most significantly in group of interstitial pneumonia, whereas FIB, D-D levels changed most significantly in the lung consolidation group. This study may help physicians to estimate the patients’ blood coagulation state in time, and give early intervention to prevent severe complications. Further studies conducted in multiple centers with functional assays are needed to validate our findings.

## Acknowledgments

We thank Assistant Professor Yongke Cao (School of Foreign Languages of Nanjing Medical University) for his kind advice in the manuscript preparation.

## Author contributions

Juan Wang conceived and designed the idea, did data collection, wrote and drafted the manuscript. Jianping Mao, Gang Chen and Yuanmei Huang did data collection. Jinjin Zhou did literature review. Changlong Gao and DantingJin reviewed the manuscript. Chenying Zhang performed the data analysis. Juan Wen and Jun Sun designed, contributed to the reviewing of the final manuscript. All authors approved the final format of the submitted manuscript.

**Data curation:** Jianping Mao, Gang Chen, Jinjin Zhou.

**Formal analysis:** Jianping Mao, Yuanmei Huang, Jinjin Zhou.

**Funding acquisition:** Juan Wang.

**Investigation:** Juan Wang, Gang Chen, Yuanmei Huang, Jinjin Zhou, Changlong Gao, Danting Jin, Chenying Zhang.

**Methodology:** Jianping Mao, Yuanmei Huang, Jinjin Zhou, Changlong Gao, Danting Jin, Chenying Zhang.

**Supervision:** Juan Wen, Jun Sun.

**Writing – original draft:** Juan Wang.

**Writing – review & editing:** Juan Wen, Jun Sun.

## Correction

When originally published, the corresponding author appeared as Juan Wen. This has been corrected to Jun Sun.
